# Three Dimensional Gait Analysis Using Wearable Acceleration and Gyro Sensors Based on Quaternion Calculations

**DOI:** 10.3390/s130709321

**Published:** 2013-07-19

**Authors:** Shigeru Tadano, Ryo Takeda, Hiroaki Miyagawa

**Affiliations:** 1 Division of Human Mechanical Systems and Design, Faculty of Engineering, Hokkaido University, Sapporo 060-8628, Japan; E-Mail: r.takeda@eng.hokudai.ac.jp; 2 Division of Human Mechanical Systems and Design, Graduate School of Engineering, Hokkaido University, Sapporo 060-8628, Japan; E-Mail: vil.shineanyallright@gmail.com

**Keywords:** gait analysis, acceleration sensor, gyro sensor, quaternion, lower limb, joint angle

## Abstract

This paper proposes a method for three dimensional gait analysis using wearable sensors and quaternion calculations. Seven sensor units consisting of a tri-axial acceleration and gyro sensors, were fixed to the lower limbs. The acceleration and angular velocity data of each sensor unit were measured during level walking. The initial orientations of the sensor units were estimated using acceleration data during upright standing position and the angular displacements were estimated afterwards using angular velocity data during gait. Here, an algorithm based on quaternion calculation was implemented for orientation estimation of the sensor units. The orientations of the sensor units were converted to the orientations of the body segments by a rotation matrix obtained from a calibration trial. Body segment orientations were then used for constructing a three dimensional wire frame animation of the volunteers during the gait. Gait analysis was conducted on five volunteers, and results were compared with those from a camera-based motion analysis system. Comparisons were made for the joint trajectory in the horizontal and sagittal plane. The average RMSE and correlation coefficient (CC) were 10.14 deg and 0.98, 7.88 deg and 0.97, 9.75 deg and 0.78 for the hip, knee and ankle flexion angles, respectively.

## 1. Introduction

Gait analysis is commonly done with an optical tracking system such as the Vicon motion analysis system (Vicon Motion Systems, Inc., Los Angeles, CA, US). Camera recordings of markers attached to a person are used to calculate three dimensional positions of body segments during gait. However, such systems are usually used indoors in controlled environments, and the camera capture range must be taken into consideration.

An alternative is to use acceleration or angular velocity data measured from small inertial sensors attached directly to the body [1–4]. This method has the advantage of identifying human motion in a wide variety of environments. This method does not directly measure position, only acceleration or angular velocity data of body segments they are attached to, therefore, a major challenge is to translate these data into meaningful three dimensional positional data, such as the joint angles of the lower limbs during gait. This can then be used for evaluating differences in the gait; for example patients with ACL related injuries [5].

In theory it is possible to integrate angular velocity or double integrate acceleration data to calculate the orientation and position of an inertial sensor. Tong and Granat calculated lower limb body segments' orientations by integrating measured angular velocity from gyro sensors strapped to the thigh and shank [6]. However, it was reported that noise in the angular velocity data resulted in integration errors. This is believed to be caused by signal noises included in the raw acceleration or the angular velocity data. Any small amount of signal noise will accumulate over time when integrated. This accumulation will cause the calculated results to deviate from the true value and is thus often referred to as drift.

As a countermeasure, some have used the cyclical properties of gait for developing signal filters and algorithms. Liu *et al.* developed a gait phase detection algorithm by using acceleration data to calculate the inclination of lower body segments during mid-stance of gait and corrected the gyro sensor drift [7]. Takeda *et al.* developed an algorithm to obtain the gravitational acceleration component from cyclic patterns in acceleration data during gait [8]. This was then used to calculate the inclination of the sensors attached to the body segments. Sabatini *et al.*, proposed using quaternions for calculating body segment orientations from angular velocity data of a body mounted gyroscope [9]. However, the proposed method used the cyclic properties of gait to compensate for drift. Favre *et al.* used acceleration data to compensate for this drift in the angular velocity data but this compensation could only be implemented in situations where gravitational acceleration is the only component measured [10].

An alternative method, not using assumptions based on the cyclical properties of gait, is to estimate the acceleration signal output at the center of rotation of the knee from two inertial sensors attached to the thigh and shank [11,12]. This method proved useful in obtaining accurate knee joint measurements and was expanded to calculating three dimensional positions of the hip, knee and ankle joint centers in a global coordinate system [13]. Though very accurate in calculating flexion-extension movements of the knee and hip as well as adduction-abduction movements of the hip, these methods did not consider the internal-external rotation of the leg.

The issue of measuring internal-external rotation of the leg is not only a problem with wearable sensors but with cameras as well. However, recently a sensor system, MTx (Xsens Technologies B.V., Enschede, Netherlands), consisting of 3D gyro sensors, 3D acceleration sensors and 3D magnetic sensors, has been made commercially available. Acceleration data and angular velocity in combination with the direction of the Earth's magnetic north is used to calculate the tri-axial orientation of the sensor, thus enabling calculation of all degrees of freedom (DoFs; in the leg including internal-external rotation. Picerno *et al.* reported the high reliability and accuracy in using such sensor systems for gait analysis with specific anatomical calibration of the lower limbs [14]. In addition, acceleration gyro and magnetic sensors proved useful in calculating the kinematics of the arm [15,16]. However, Brodie *et al.* reported that when compared with a camera based analysis, three dimensional orientation accuracy errors existed, even during static states [17]. Avoiding such errors required recalibration of the sensors on a regular basis, and required a homogeneous magnetic environment which is not realistic for monitoring gait in everyday situations.

The work presented here is a pilot study on a new theoretical approach for calculating the 3-dimensional orientation of various body segments of the lower limb during gait using quaternions. The proposed approach developed a calibration method for deriving the relationship between the sensor coordinate system and the body segment coordinate system to minimize sensor attachment errors. In addition, the unique properties of quaternion allowed 3-dimensional lower limb orientation calculations, not possible in previous studies [11–13]. The method only required the acceleration and angular velocity data measured from wearable sensor units, and did not rely on external sources such as magnetic fields. Five healthy volunteers participated in the experiment and their gait motion was measured using the proposed method and a commercially available motion analysis system.

## 2. Method

This method defines the gait posture as a product of initial posture and the subsequent angular displacement. First, the direction of gravitational acceleration, measured by acceleration sensors, was used to calculate initial posture of body segments. Then, the angular displacement after initial posture was estimated using angular velocity measured by gyro sensors. Signal processing was implemented on the raw angular velocity data to remove noise, consequently reducing drift. Here, the angular displacement was expressed using a quaternion based posture expression. Finally, the characteristic gait motion established by this work was displayed using a three dimensional wire frame model.

### 2.1. Sensor System

Seven sensor units (WAA-006, Wireless Technologies, Inc., Tokyo, Japan) were used for measuring linear acceleration and angular velocity. Each sensor unit was 39 mm × 44 mm × 12 mm in dimension, weighed 20 g and could operate for 6 hours on a rechargeable internal lithium battery. The measurement range of the acceleration sensor was ±2 G/4 G in all axes with accuracies of 0.001 G/0.002 G respectively. The gyro sensor range was ±500 deg/s for the X, Y axes with accuracies of 1.46 deg/s, and ±300 deg/s for the Z axis with an accuracy of 4.37 deg/s. The maximum sampling rate was 500 Hz and the measured data could be transferred wirelessly (Bluetooth ver.2.0 + EDR) to a laptop computer in real time.

### 2.2. Wire Frame Gait Model

A wire frame model of a volunteer is created to visually confirm the lower limb motion of gait and calculate joint angles. The model uses the orientations of each body segment and specific body measurements such as; bicristal breadth, iliospinale breadth, iliospinale height, tibiale height and sphyrion height. Sensor units are placed on seven predefined segments of a volunteer: pelvis (PE; posterior center point between the left and right iliac crest) both thighs (RT, LT; top of the center of the quadriceps), both shanks (RS, LS; anterior side of the tibia bone), both feet (RF, LF; dorsum side of the foot). These locations were chosen to minimize the effects of muscle movement to the sensor units.

**Figure 1.**
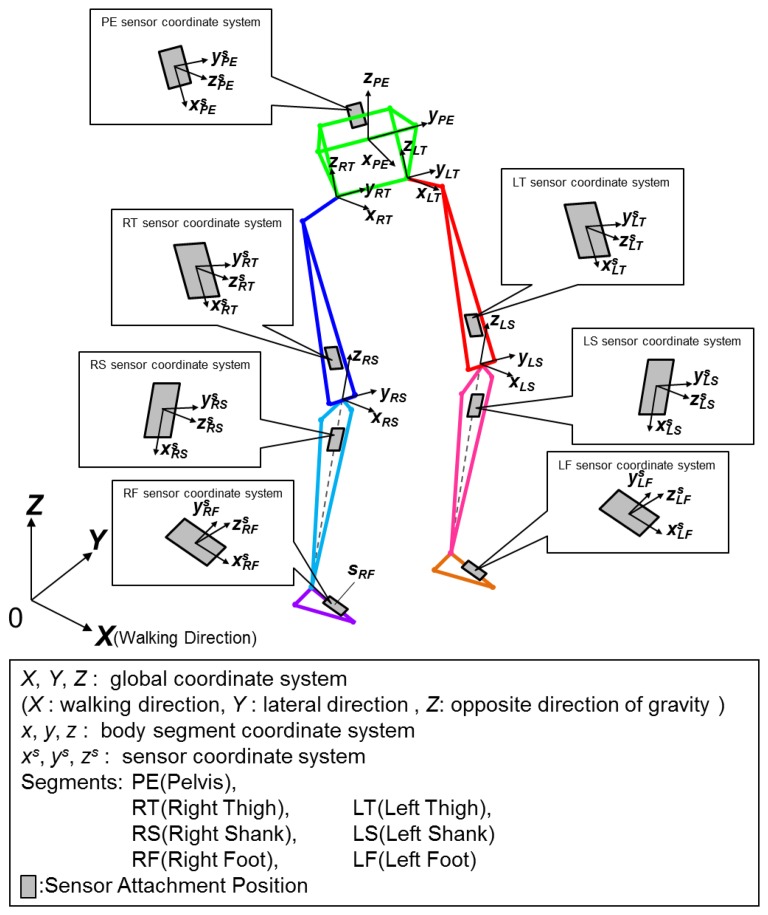
Gait wire frame model and coordinate systems. The *X*, *Y*, *Z* coordinates represent the global coordinate system, where the *X* axis is the walking direction, the *Y* axis is the left-lateral direction, and the *Z* axis the direction opposite to gravity. PE, RT, LT, RS, LS, RF and LF represent the lower limb body segments. *x*, *y*, *z* represents the body segment coordinate system and *x^s^*, *y^s^*, *z^s^* represents the sensor coordinate system.

Three different coordinate systems were used (Figure 1). First, the sensor coordinate system represents the three orthogonal axes in which the acceleration and angular velocity are measured. Second is the segment coordinate system for the body segments that each sensor is attached to and last is the global coordinate system.

### 2.3. Calibration of the Coordinate Systems

**Figure 2.**
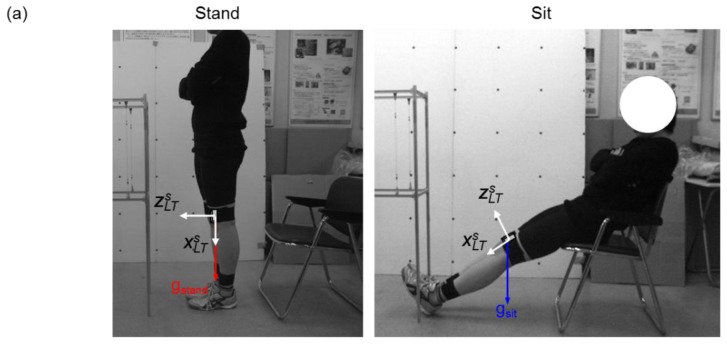

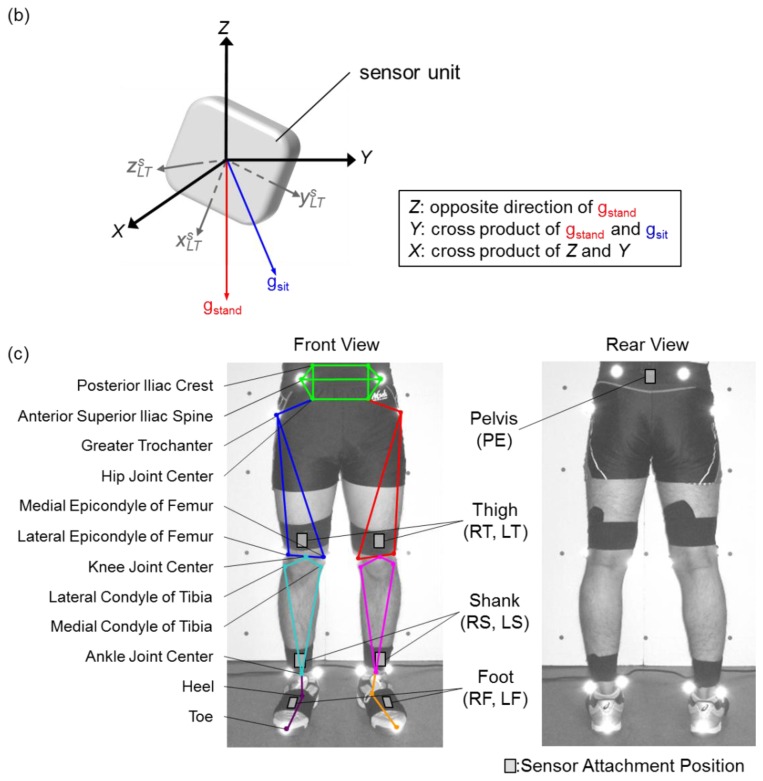
Sensor and global coordinate system calibration for LT. (**a**) Gravitational acceleration g_stand_ and g_sit_ are measured from acceleration sensors attached to a volunteer from two different postures. (**b**) The axes for the global coordinate system are defined as: *Z* the opposite direction of g_stand_, *Y* the cross product of g_stand_ and g_sit_ and *X* the cross product of *Z* and *Y*. (**c**) Sensor attachment location and gait wire frame model. The wire frame model is created by connecting characteristic positions of the lower limb. Sensor units are attached to seven body segments of the lower limb.

A calibration procedure for establishing the relationship of the sensor coordinate system to the body segment coordinate system was conducted. Previously, Dejnabadi *et al.* used still camera images and markers, attached to anatomically characteristic locations of the lower limb, to estimate the coordinates of wearable sensors [18]. This work used a similar but more refined procedure to obtain the rotation matrix for converting the sensor to body segment coordinate system. The procedure was conducted as follows:

First, the volunteers were asked to do a static calibration procedure of standing upright and sitting with outstretched legs where the gravitational acceleration vector **g_stand_** and **g_sit_** for the respective positions were measured (Figure 2a). In order to conduct a calibration for all sensors at once, the volunteers were instructed to stand up straight with their toes aligned and their feet in parallel for the standing posture. This procedure was conducted to align all the sensor units in 2-D sagittal plane and minimize their attachment errors, caused by misalignments of coordinate systems between the sensor and body segments. For the sitting posture, volunteers were asked to put their hips on the edge of their chair while maintaining their knee and ankle joint angle the same for the standing posture. Since **g_stand_** and **g_sit_** will differ in the sensor coordinate system between the two positions, as shown in Figure 2b, the axes for the global coordinate system were defined as: Z axis being the opposite direction of **g_stand_**, Y axis the cross product of **g_stand_** and **g_sit_**, X axis as the cross product of Z and Y. As a result, a rotation matrix ***R****_SG_* for converting the three orthogonal axes of the sensor coordinate system to the global coordinate system was obtained.

Second, measurements of the lower limbs were required to calculate the body segment coordinate system. Reflective markers were attached to anatomical characteristic positions of the lower limbs, and still digital images were taken from the front and side of the volunteer (Figure 2c). The 18 anatomical locations used for this method were: Posterior iliac crest (2), anterior superior iliac spine (2), greater trochanter (2), lateral epicondyle of femur (2), medial epicondyle of femur (2), lateral side of ankle (2), medial side of ankle (2) and heel and toe (4). From the images, another rotation matrix ***R****_GB_* for converting the global to the body segment coordinate system was defined.

Finally by using the two rotation matrices, a rotation matrix ***R****_SB_* for converting the sensor to body segment coordinate system was established:

(1) $$R_{SB}=R_{SG}R_{GB}$$

### 2.4. Orientation Calculation

The orientation of a sensor unit *θ_t_* can be calculated by adding the initial orientation *θ*_0_with the subsequent angular displacement *ω*. Therefore, the following equation can be used:

(2) $$\theta_{t}=\theta_{0}+\int_{0}^{t}\omega dt$$

#### 2.4.1. Initial Sensor Orientation Using Acceleration Data

The initial sensor orientation is calculated using the acceleration data as a measure for inclination. The acceleration measured by an acceleration sensor can be expressed by the following:

(3) $$S_{i}=\alpha_{i}-g_{i}$$

*S* being the sensor output, *α* the translational acceleration and *g* the gravitational acceleration along axis *i* (*i* = x, y, z). During a static state, such as standing upright, *α* is 0 and only *g* is present:

(4) $$\theta_{i0}=\arccos\frac{g_{i}}{\left\|g\right\|}$$

Equation (4) can be used to calculate the initial inclination *θ_i_*_0_ of axis *i*, thus giving initial orientation (***g*** is the resultant vector of gravitational acceleration).

#### 2.4.2. Angular Displacement Measurement Using Quaternion

Three dimensional rotation using quaternions can be expressed as follows [19]:

(5) $$q=\cos\frac{\phi }{2}+n\sin\frac{\phi }{2}$$

(6) $$r^{\prime }={qrq}^{*}$$

Here *q* is the quaternion and ∅ the rotation of angle around unit vector ***n***. q* is the conjugated quaternion of *q* and ***r'*** is the vector after the rotation applied to ordinary vector ***r***.

In this work, the rotation around a given axis will be calculated from the angular velocity:

(7) ϕ=‖ω‖Δt

In Equation (7), it is presumed that ***ω*** is the angular velocity vector calculated from the gyro sensor data at time Δ*t*. If this is inserted into Equation (5) we get the following:

(8) $$q=\cos\frac{\left\|\omega \right\|\Delta t}{2}+\frac{\omega }{\left\|\omega \right\|}\sin\frac{\left\|\omega \right\|\Delta t}{2}$$

By continuously calculating *q* for each sampling rate after the initial orientation, the angular displacement is obtained.

#### 2.4.3. Noise Reduction and Signal Filtering

One of the major drawbacks of integration of angular velocity data from gyro sensors is drift. The noise included in the raw gyro sensor data accumulates over time with integration. In order to quantify the amount of drift, a simple bench-top procedure was conducted on the sensors.

**Figure 3.**
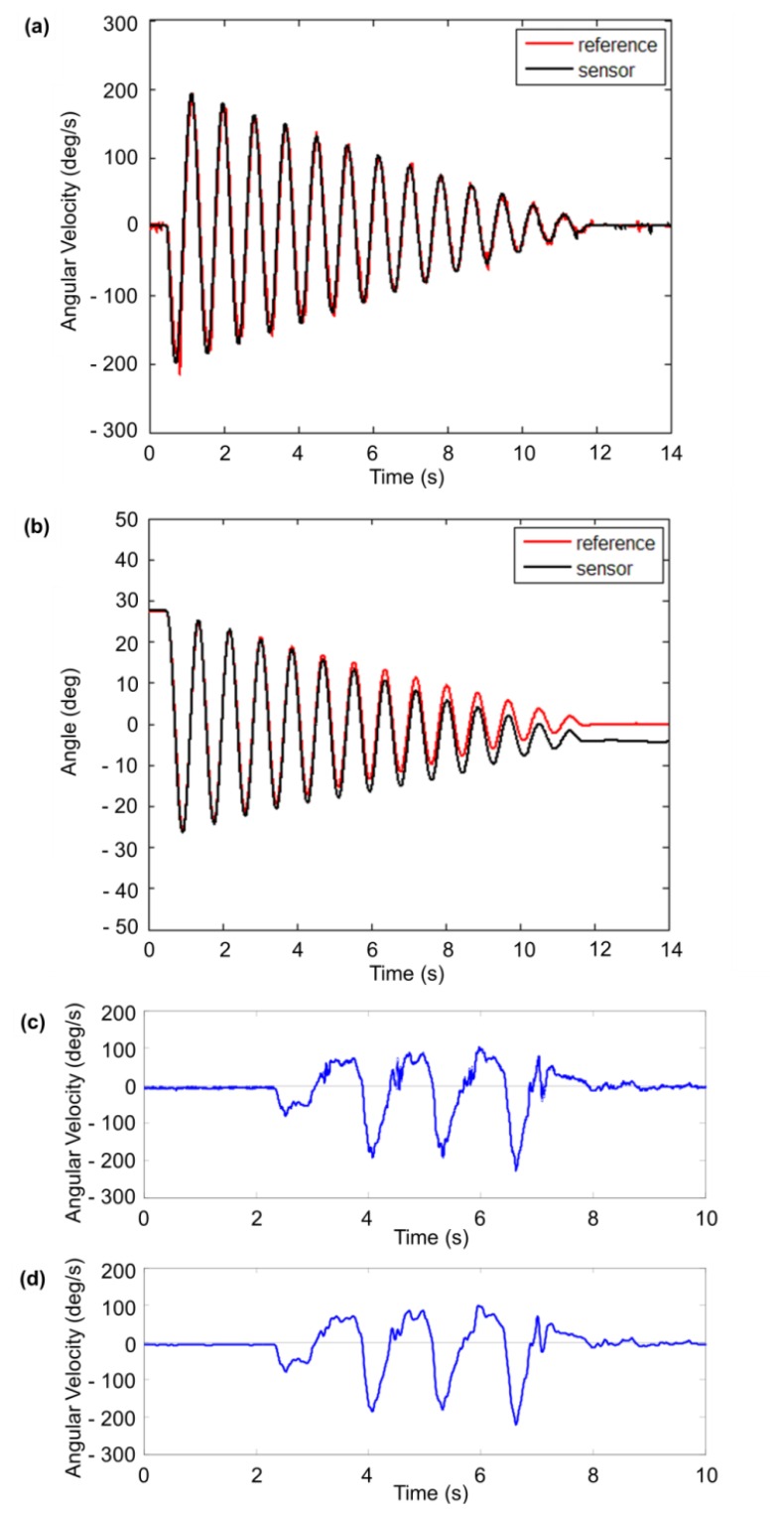
Sensor calibration results and signal filtering for noise reduction. (**a**) Comparison of the angular velocity results calculated from the gyro sensor and the reference CCD camera for a single link pendulum test. The vertical axis represents angular velocity while the horizontal axis represents the time. (**b**) Comparison of the pendulum link angle between gyro sensor and the CCD camera. The vertical axis represents angle while the horizontal axis represents the time. (**c**) A raw gyro sensor before any signal filtering. (**d**) The same gyro sensor data after applying an IIR digital Butterworth filter. The vertical axis represents angular velocity while the horizontal axis represents the time for both (c,d).

The bench-top calibration procedure involved a single link pendulum with the wearable sensors and optical tracking markers attached to the swing link. An infrared CCD camera was used to record the optical tracking markers to give the reference angle of the pendulum. The angular velocity and pendulum angle was calculated and the results are shown in Figure 3a.

The angular velocity showed high agreement with the reference (correlation coefficient (CC): 0.98, RMSE: 0.31 deg/s). However, when integrating the angular velocity of the gyro sensors to calculate the pendulum angle, angular drift was present (Figure 3b). The angle drifted 4.47 deg in 14 s and the RMSE was over 2.67 deg. Even though the measurement time was short, drift had appeared.

In this work, an IIR (infinite impulse response) digital 4th order Butterworth filter was used to remove noise from the raw gyro sensor data. This low pass filter was implemented using MATLAB, where the cutoff frequency was set to 12 Hz through experimental rule. The filter was applied in both the forward and backward direction to cancel out any phase lag. In addition to this, an offset value was found in the gyro sensor data. When the sensors were kept at a static state, the measured data showed values near but not exactly at zero. Therefore, the mode value of the gyro sensor data at a static state was calculated and subtracted from the measured data for each individual axis of each sensor unit. The effect of this noise reduction and signal filtering is shown in Figure 3c,d.

### 2.5. Visualization of Gait Motion

**Figure 4.**
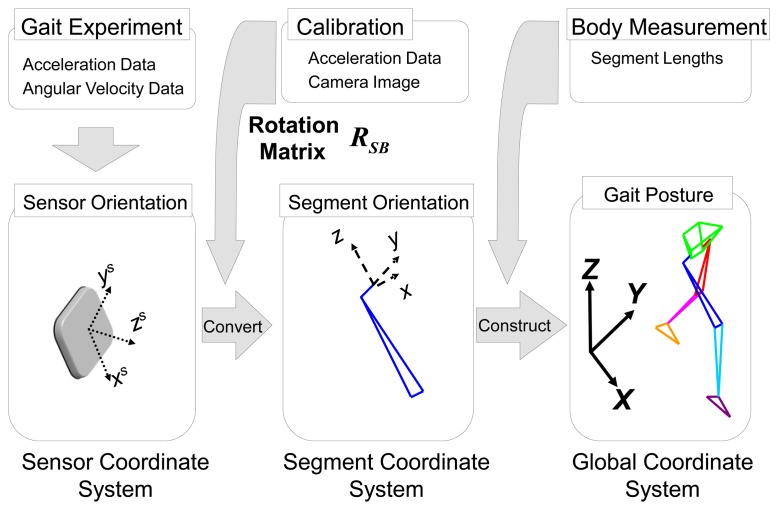
The process flow of gait experiment for visualization of gait motion using a gait wire frame model. Acceleration and angular velocity data from the sensor coordinate system are converted to be expressed in the body segment coordinate system using rotation matrix R_SB_. Gait motion on the wire frame model is constructed by segment orientation and length.

An animation was created to visualize gait motion using the wire frame model for both this method and the camera based method for comparison. The process flow of this visualization is shown in Figure 4.

Firstly, the gait experiment provided the acceleration and angular velocity required for calculating the sensor orientation. Secondly, the rotation matrix ***R****_SB_*, obtained using the method in Section 2.3, was used for converting the sensor orientation into the body segment coordinate system. Finally, the gait motion, calculated from the method described in Section 2.4, along with the individual body measurements of a volunteer, will let us create a moving lower limb wire frame model in the global coordinate system. The anatomical characteristic points as seen in Figure 2c were used in this process. After the visualization of gait was completed for this method, a similar model was created by using the camera based tracking method as well.

## 3. Experiments

Experiments were conducted indoors on a straight flat floor with five healthy volunteers with no history of physical disabilities or injuries. All volunteers gave informed consent for participation in the gait experiment and the information of each volunteer is shown in Table 1. The volunteers were asked to perform the calibration procedure of Section 2.3, then afterwards do one gait trial consisting of: standing still, walking in a straight line and then standing still again.

Elastic bands were used to fix the sensor units on top of the skin of seven body segments. Reflective markers were placed on 18 anatomical characteristic positions (the same position as those mentioned in Section 2.3.) of the lower limb and a camera based tracking system (DIPP-Motion Pro, Ditect Co., Ltd., Tokyo, Japan) was used to record the volunteer's motion. This system consisted of six high speed CCD cameras connected to a workstation with motion analysis software [8,10,20]. The software included in this system automatically detects and gives the 3-dimensional coordinate data of each marker. These coordinate data were then used to create a comparative wire frame model and calculate the joint angles. The cameras were operating at 30 fps, and walking distance was restricted to approximately 5 m due to the range of the cameras. The measurements from the cameras and the sensor units were synchronized using the acceleration data by detecting the heel contact timing of the first step of the volunteers' gait.

## 4. Results

**Figure 5.**
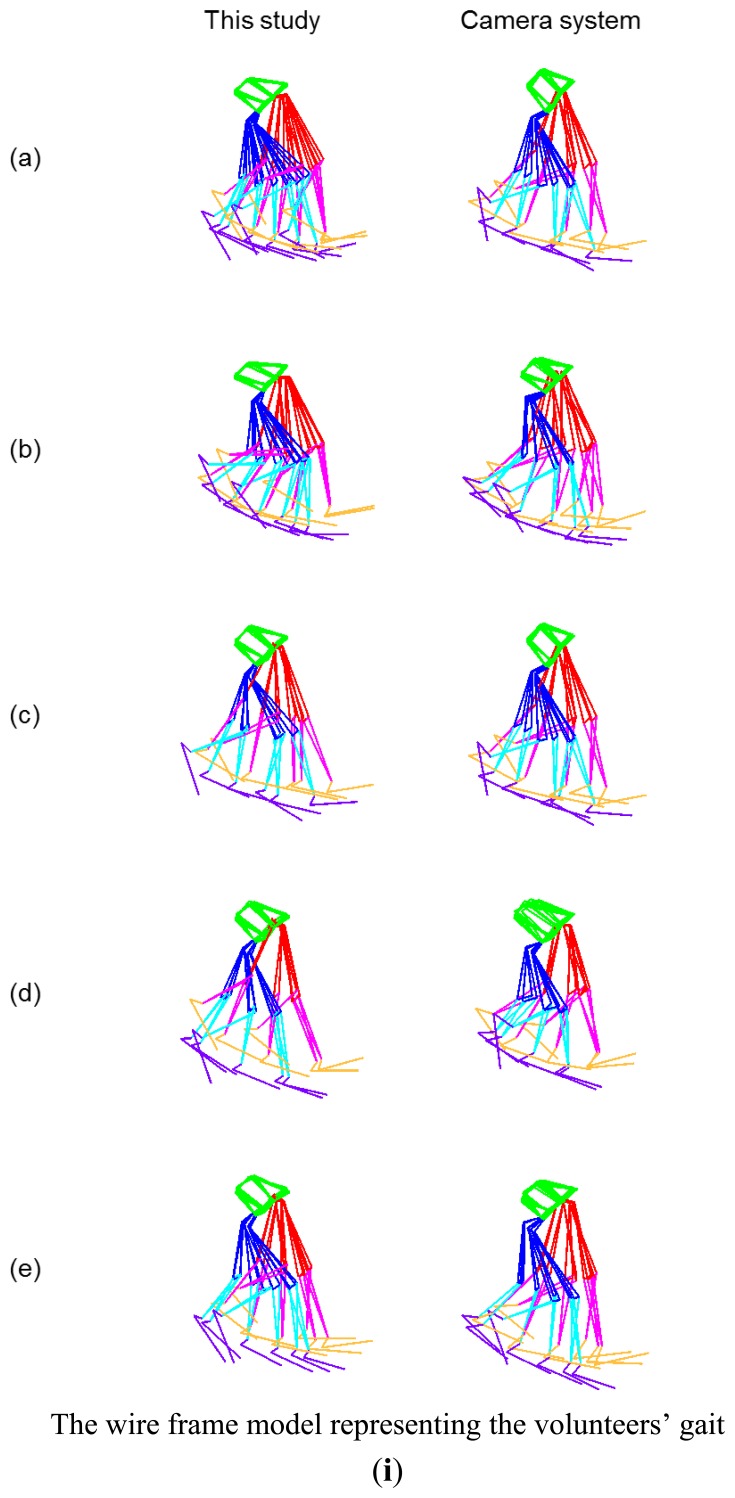

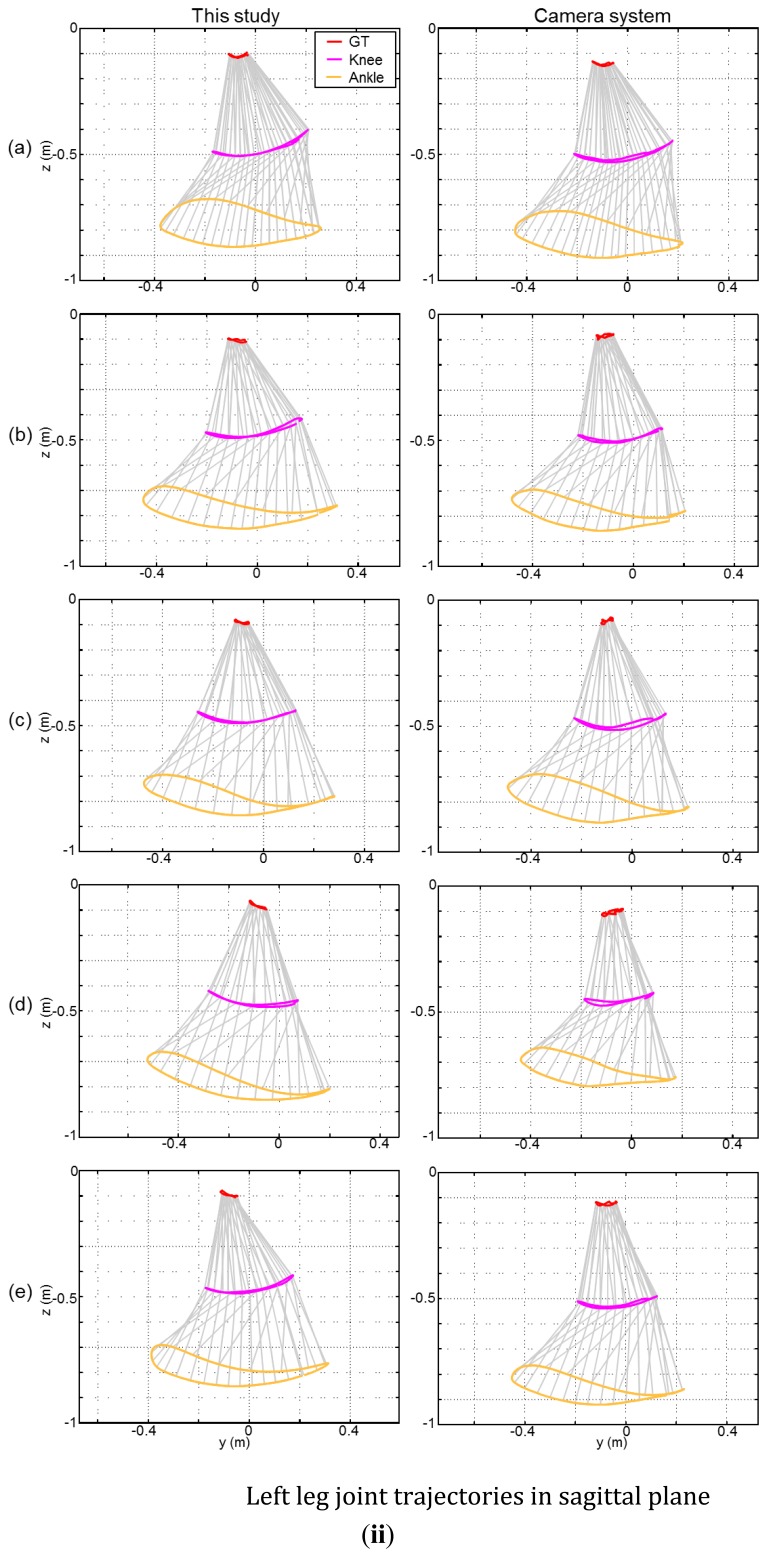

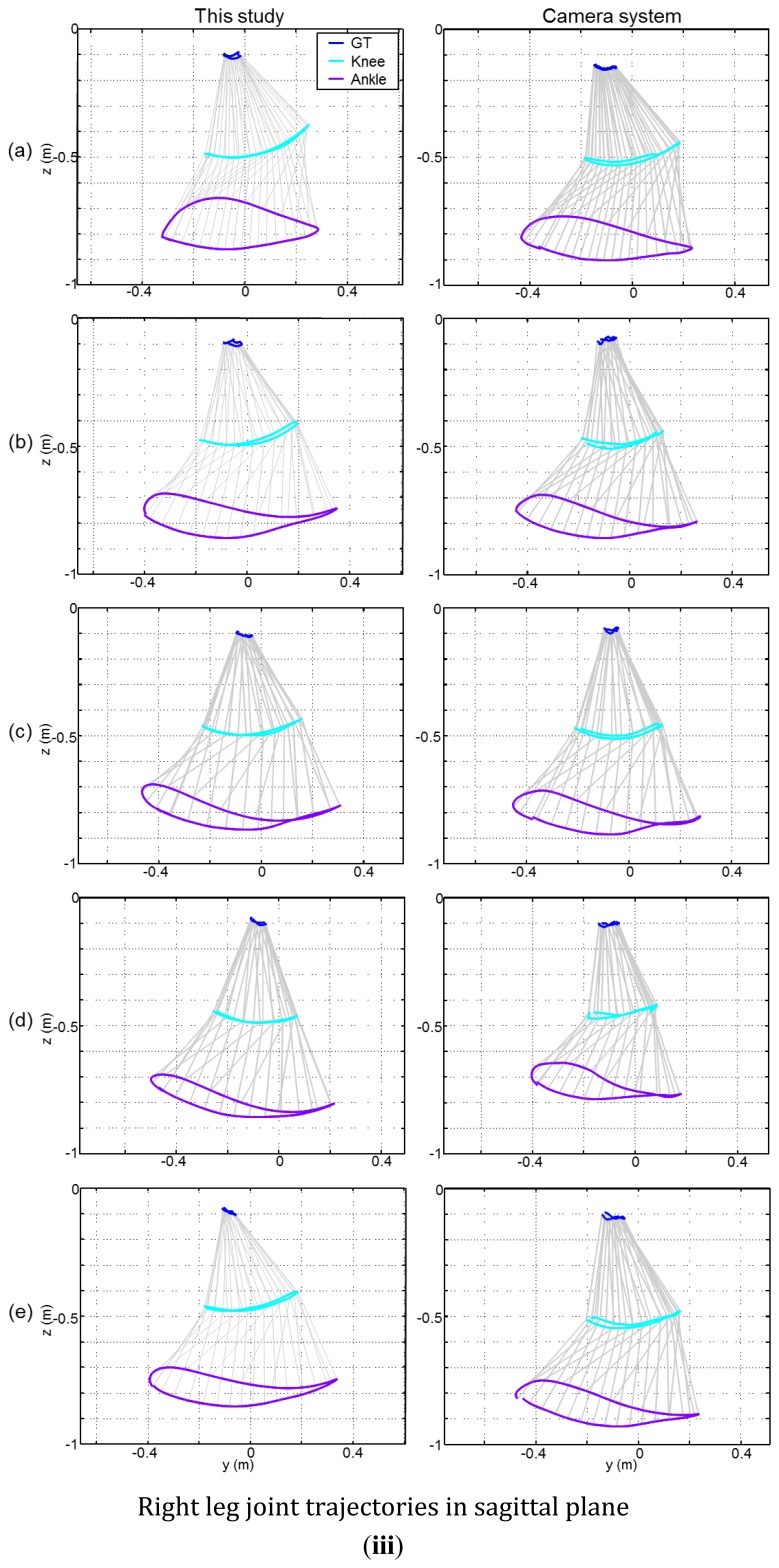

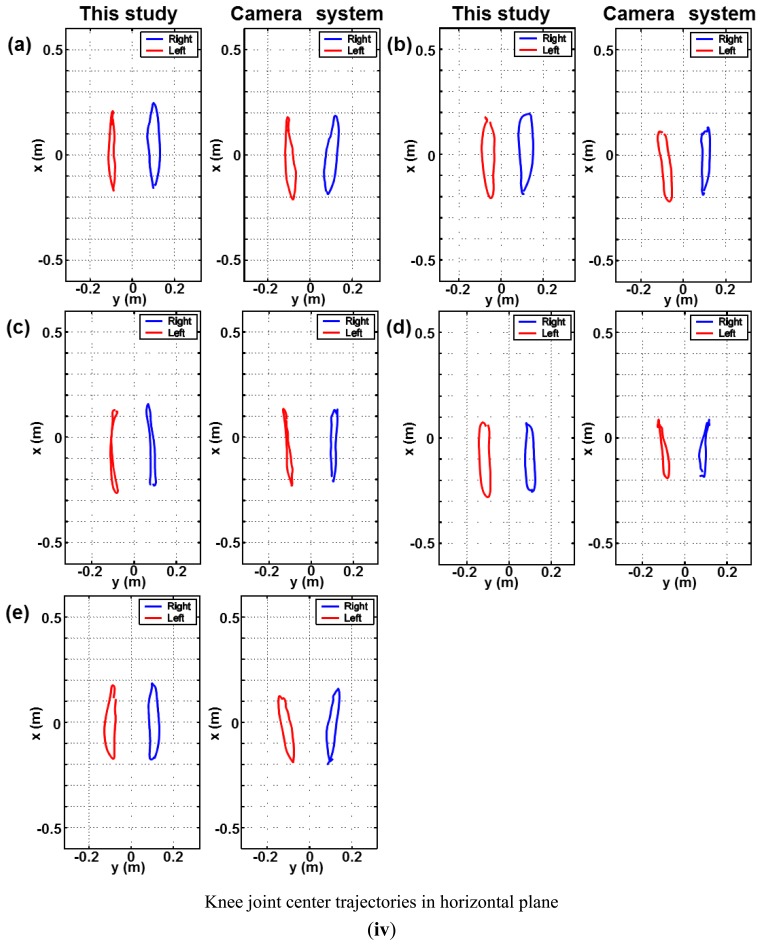

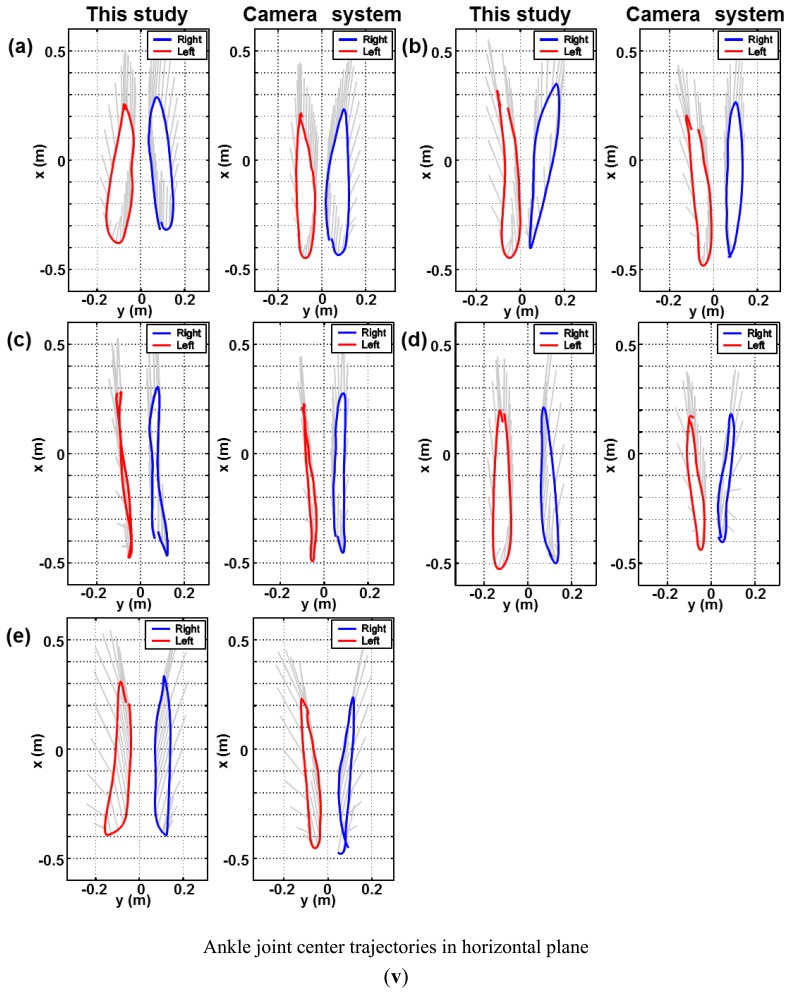
The figures on the left are the results obtained using this method and the right are those of the camera system. (**i**) The wire frame model representing gait of volunteers (a–e). The plot of the greater trochanter (GT), knee joint center and ankle joint center for the right leg (**ii**) and left leg (**iii**) during one gait cycle in the sagittal plane. The vertical axis represents the *z* axis and the horizontal axis represents the *x* axis in the global coordinate system. The plot of the knee joint center (**iv**) and ankle joint center (**v**) during one gait cycle in the horizontal plane. The vertical axis represents the *x* axis and the horizontal axis represents the *y* axis in the global coordinate system. The left knee is shown in red and the right in blue, the grey lines emanating from these lines represents the direction of the foot segment with respect to the ankle joint.

The wire frame representations of a volunteer's gait using this method and camera system are shown in Figure 5i. The wire frame models show an overlapping plot at 5 Hz intervals. The wire frame representations of both methods are quite similar, however differences can be seen in the pelvis segment where the camera method is tilted more inwards. The amount of overlap is greater in this method due to longer measurement time than the camera.

The hip joint center estimation was based on the method by Leardini *et al.* [21], the knee joint center was assumed to be the midpoint between the lateral and medial epicondyles, and the ankle joint center was assumed to be the midpoint between the lateral and medial ankle anatomical landmarks.

**Figure 6.**
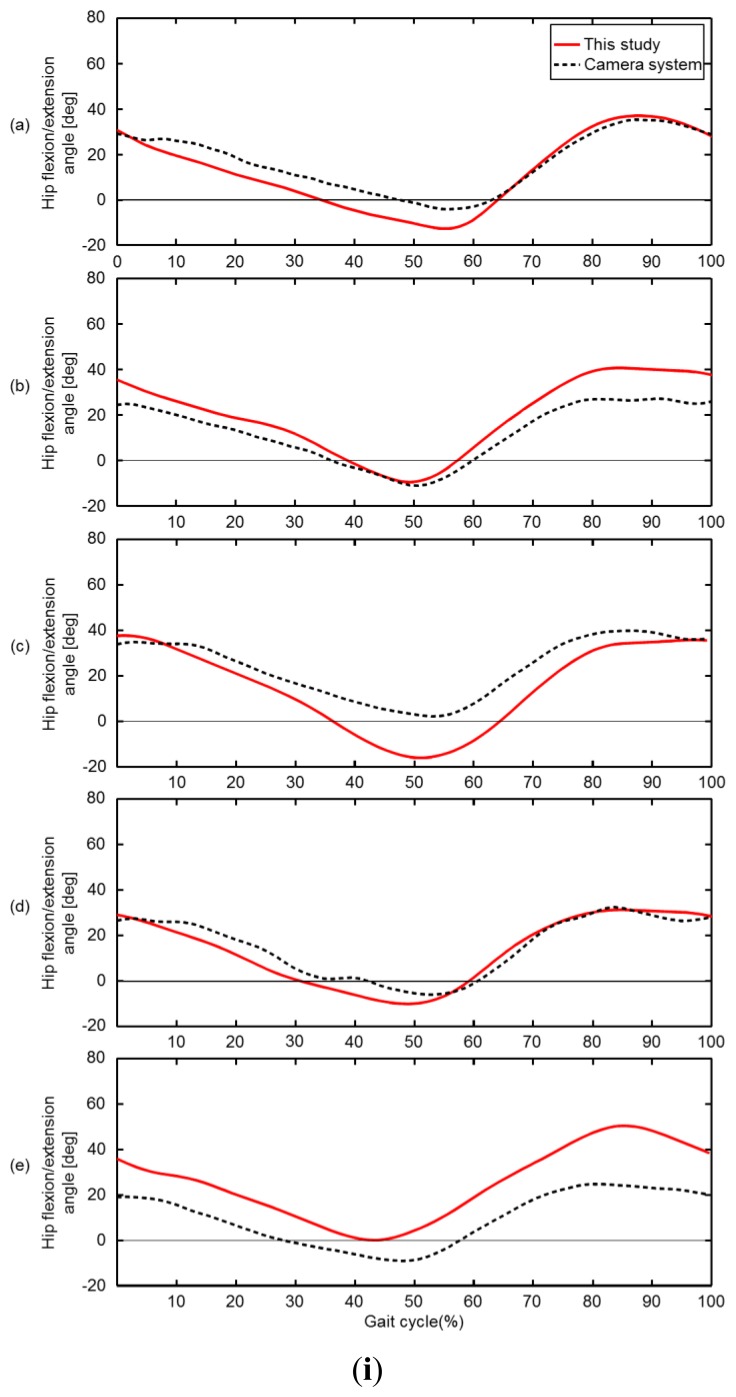

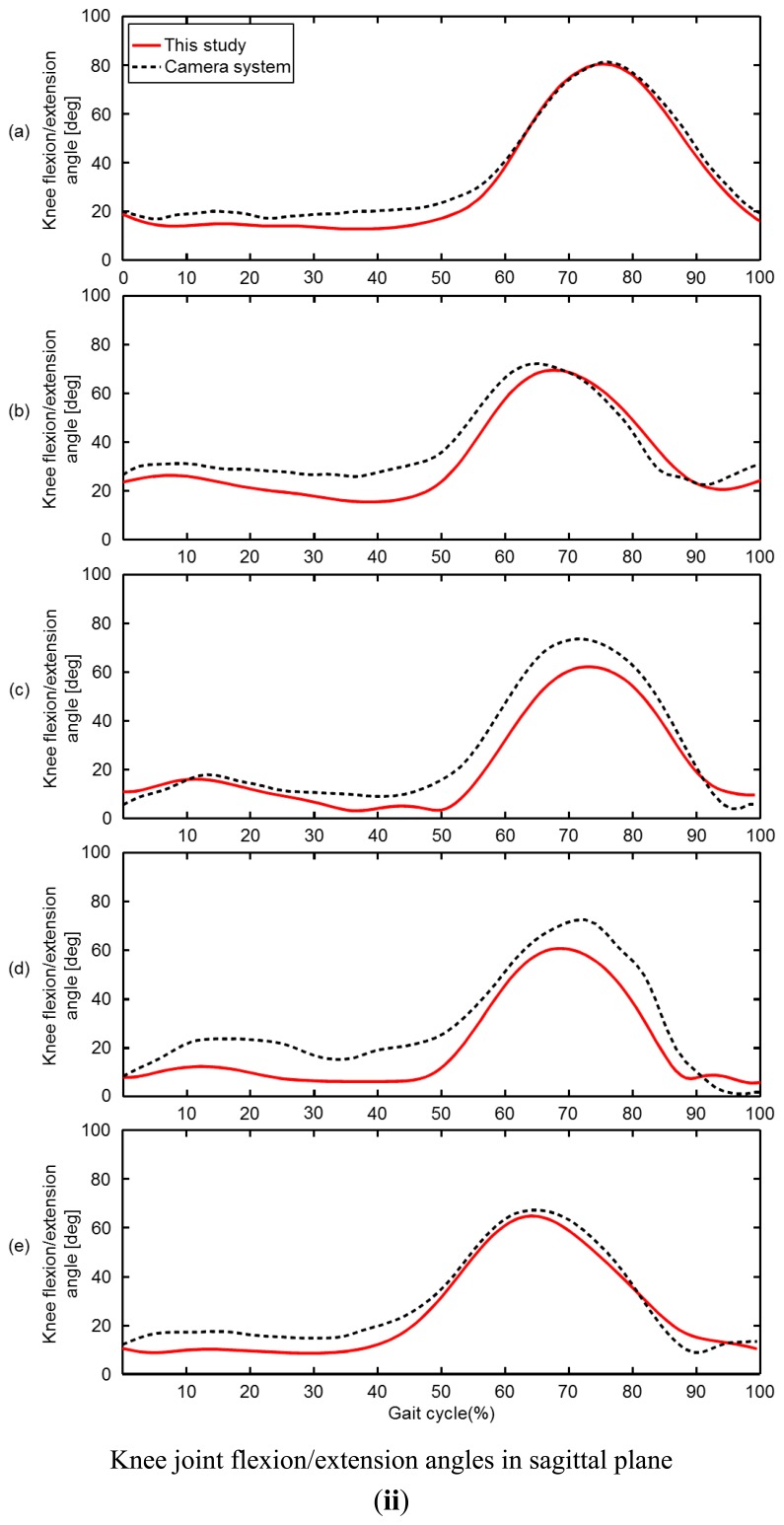

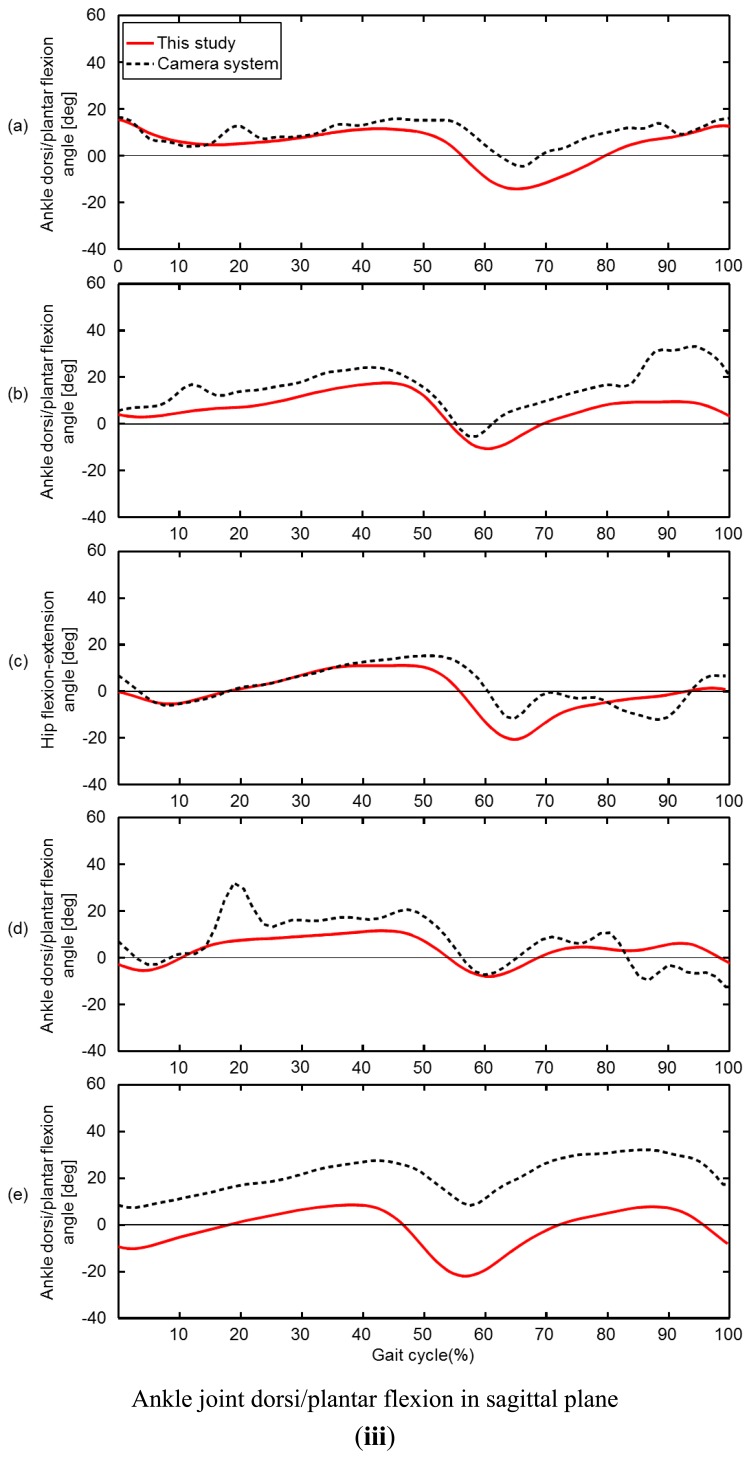
Comparison of the hip flexion/extension angle (**i**), the knee flexion angle (**ii**) and ankle dorsi/plantar flexion angle (**iii**) during one gait cycle for volunteers (a–e). The red line is the result obtained from this study and the dotted line is the results from the camera. For (i) and (ii) the vertical axis shows the flexion/extension angle, where 0 is the angle in the upright position and flexion is positive. For (iii) the vertical axis shows the dorsi/plantar flexion angle, where 0 is the angle in the upright position and dorsiflexion is positive. The horizontal axis shows the percentage of one gait cycle.

Figure 5ii,iii show the comparison between this method and camera system for the trajectories of the left and right great trochanter, knee joint center and ankle joint center in the sagittal plane. The results of this method seems to be shifted more to the positive z direction than the camera method, however the overall tendency is basically the same. In addition, the trajectories of this method show that the results for the right and left leg are symmetric. The comparison for the knee joint center and ankle joint center trajectories projected on the X-Y plane are shown in Figure 5iv,v, respectively. Both sagittal and horizontal trajectories are of one gait cycle plotted at 20 Hz. The results of this method seems to be shifted more to the positive x direction than the camera, however the length and width of the trajectories are almost the same. The knee and ankle joint angles during one gait cycle, for both camera and this method, are shown in Figure 6, respectively. The vertical axis represents angles in degrees while the horizontal axis represents the percentage of one gait cycle.

Table 2 shows the root mean square error (RMSE) and correlation coefficient (CC) between the joint angles calculated using this method and that of camera system. The results are for all five volunteers and for one gait cycle of each leg. The average RMSE and CC for each joint of both legs were; hip = 10.14 deg and 0.98, knee = 7.88 deg and 0.97, ankle = 9.75 deg and 0.78 respectively.

## 5. Conclusions and Outlook

High CC was observed for all volunteers, however there was variation in the RMSE. High CC can be interpreted as the method presented here being high in reliability. The variation in the RMSE is believed to be caused by inaccuracies in calculating the lower limb measurements from camera images. This is because the calculating rotation matrix ***R****_SB_* relies on still camera images and acceleration data only. The underlining assumption in this calibration was that the sensors will only rotate orthogonally to the sagittal plane of the volunteer during the sitting and standing positions. If any other rotations, such as internal-external rotation of the hip and knee, are different in the two postures it would result in errors in the calibration.

Another observation is that the CC for the ankle joint was lower than those of the hip or knee joint. There are two possible reasons for this: inaccuracies in the camera or the sensors. Since the accuracy of camera based tracking relies on the pixel resolution, the bigger the field of view the more information one pixel will hold, thus lowering the measurement accuracy. In addition, often the most distal segments of the limb move the most during gait, therefore they are more likely to have the images distorted when they are photographed at the edge of the lens. Another, reason may be because the camera based motion tracking was done at 30 Hz, which may have been too low.

Though not present in the figures, the angular velocities were more problematic as the sensors were placed at the distal parts of the limbs. This is due to the fact that as the angular velocities detected on the RF and LF were much higher than those attached to the RT, LT, RS, LS velocities. Therefore RF and LF are more vulnerable to errors during gait motion. The larger angular velocity detected on the RF and LF are believed to be caused by the heel contact and toe off timing of the stance phase. During heel contact the ankle quickly does a planar flexion motion to flatten out the foot. During toe off the ankle again does a quick planar flexion motion to push the foot off into the swing phase. These results seem to concur with previous findings, where it was reported that the foot angular velocity was higher than those of the shank or the thigh [22,23]. This effect can also be seen in the differences in the ankle joint trajectories in the horizontal plane in Figure 5v.

One of the improvements that the implementations of quaternions allowed for was the representation of three dimensional rotations while avoiding singularities. Previously, the calculation of internal-external hip joint rotation was not possible [8,13]. Therefore, quaternions allowed for a more accurate representation of joint motion. Instead of directly comparing the internal-external rotation angles of each joint between this method and the camera, the authors believed that the horizontal trajectory representation will be more useful in recognizing the abnormalities in gait. The results from Figure 5iv,v shows that this method can even detect differences in the joint trajectories between the left and right leg of healthy volunteers. For example, the onset of knee osteoarthritis (OA) can change the varus-valgus characteristic during gait [24,25]. Since varus-valgus motion in the knee will affect the horizontal trajectory of the ankle, this system may enable clinicians to quantify the severity of OA.

For clinical purposes it is preferable to make the results available in real time such as those proposed by Kavanagh *et al.* [26]. However, currently the proposed method takes up to 2 hours to complete. In the future, we would like to shorten this time by pruning and simplifying the experimental procedures. The gait trials in this work were limited to only 5 m due to the capture range of the cameras. The work presented here is still in the pilot stages and the 5 m trials were only intended to verify how this method compares to a reference camera based capture system.

This method has implemented sensor attachment error calibration (Section 2.3) and signal filtering (Section 2.4.3) to minimize the effects of sensor drift. However with all these precautions in place, there were differences to the camera based results. In particular the ankle joint angles showed significantly lower CC and larger RMSE. This paper was not intended to propose a method for completely removing the sensor drift, but to just minimize its effects. Since gait trials reported here are only conducted for 5 m, the drift effect might not have been apparent. Application to longer distance gait trials is still unknown and this underlying limitation will have to be investigated to better assess the accuracy and repeatability. Recently, there has also been a trend to use state observers and Kalman filters in combination with inertial sensors for motion analysis [27]. The key to further increasing the measurement accuracy and shortening the calculation time may also lie here, however these methods require a good model of the expected motion to produce reliable results.

Even though Vicon motion analysis systems are considered the gold standard for 3-D motion tracking, there are many papers reporting its accuracy errors [28,29]. Thus, the necessity of investigating the absolute accuracy for a wearable sensor system was in question. It is true that a wearable sensor system also suffers from many factors, such as: sensor attachment errors, regular calibration maintenance, external signal noise, signal filtering errors and integration drift. Even the commercially available MTx system is reported to have significant errors [30,31]. Therefore wearable sensor systems may never become more accurate than a camera based system. The objective of this study was to provide a fairly simple and relatively accurate method for quantifying gait. The proposed method showed that it can provide quantitative information such as differences in left/right hip, knee or ankle joint trajectories and the flexion/extension joint angles during a gait cycle. In addition, the gait wire frame model and the horizontal knee and ankle trajectories can identify abnormalities during gait. Therefore, the next step is to evaluate the potential of the proposed method in clinical applications with a larger number of volunteers.

## Figures and Tables

**Table 1.** Information of the volunteers. Age, gender, past injuries, height, weight, bicristal breadth, iliospinale breadth, iliospinale height, tibiale height and sphyrion height are listed.

**Volunteer**	**(a)**	**(b)**	**(c)**	**(d)**	**(e)**
Age	24	23	22	27	23
Gender	Male	Male	Male	Male	Male
Past injuries	None	None	None	None	None
Height (cm)	180.0	169.0	172.0	170.0	178.0
Weight (kg)	67.0	62.0	61.0	62.0	69.0
Bicristal breadth (cm)	29.7	28.9	29.9	29.9	29.6
Iliospinale breadth (cm)	27.5	27.3	28.2	28.1	28.0
Iliospinale height (cm)	98.5	92.0	96.0	92.2	103.3
Tibiale height (cm)	46.5	45.5	44.1	42.0	46.7
Sphyrion height (cm)	6.0	5.0	6.9	6.2	6.8

**Table 2.** Analytical results of the joint angles obtained from this study and camera system. The root mean square error (RMSE) in degrees and correlation coefficient are shown for the resultant angles of the hip, knee and ankle joint angles. The results are for all five volunteers and for one gait cycle of each leg.

**Volunteer**	**Joint**	**Left Leg**		**Right Leg**	
	
		**RMSE (deg)**	**CC**	**RMSE (deg)**	**CC**
(a)	Hip	6.00	0.98	6.30	1.00
	Knee	4.10	1.00	9.80	1.00
	Ankle	6.90	0.83	9.60	0.85

(b)	Hip	8.20	0.99	14.60	0.96
	Knee	7.80	0.95	8.60	0.93
	Ankle	11.20	0.75	4.10	0.89

(c)	Hip	10.60	0.98	8.10	0.96
	Knee	8.00	0.98	5.20	0.98
	Ankle	6.60	0.76	8.10	0.44

(d)	Hip	4.20	0.97	6.80	0.99
	Knee	10.70	0.96	11.20	0.97
	Ankle	8.40	0.70	5.80	0.88

(e)	Hip	15.90	0.98	20.70	0.99
	Knee	4.90	0.98	8.50	0.98
	Ankle	23.40	0.83	13.40	0.83

Average	Hip	8.98	0.98	11.30	0.98
	Knee	7.10	0.97	8.66	0.97
	Ankle	11.30	0.77	8.20	0.78
